# Illness uncertainty in individuals with gynecologic and breast cancer: A latent profile analysis and structural equation modeling

**DOI:** 10.1371/journal.pone.0358366

**Published:** 2026-09-18

**Authors:** Hui Zeng, Yan Lu, Quanping Zhao, Jingjing Gong, Li Mao, Yuxuan Wei, Xiaodan Li

**Affiliations:** 1 Department of Obstetrics and Gynecology, Peking University People’s Hospital, Beijing, China; 2 College of Nursing, Hebei University, Hebei, China; 3 Department of Breast Surgery, Peking University People’s Hospital, Beijing, China; Kyung Hee University, KOREA, REPUBLIC OF

## Abstract

**Background:**

Gynecologic and breast cancers pose a significant global health burden for women. Illness uncertainty is a common psychological challenge among patients with gynecologic and breast cancers, particularly in settings with limited supportive care resources. This study aimed to identify latent classes of illness uncertainty, examine their associated factors, and investigate its mediating role between social support and depressive symptoms.

**Methods:**

A cross-sectional study was conducted from December 2024 to June 2025, enrolling 413 patients from a tertiary hospital in Beijing using convenience sampling. Data were collected with a general information questionnaire, the Mishel Uncertainty in Illness Scale (MUIS), the Social Support Rating Scale (SSRS), the 9-item Patient Health Questionnaire (PHQ-9), and the Generalized Anxiety Disorder-7 scale (GAD-7). Latent profile analysis was applied to identify subgroups of illness uncertainty. Univariate analysis and multinomial logistic regression were used to examine influencing factors. Structural equation modeling was employed to test the mediating effect.

**Results:**

Three latent classes were identified: low uncertainty-psychological adaptation (8.0%), moderate uncertainty-complexity distress (37.6%), and high uncertainty-cognitive ambiguity (54.4%). Educational level, caregiver type, time since diagnosis, social support, and depressive symptoms were significantly associated with class membership. Mediation analysis revealed that illness uncertainty partially mediated the relationship between social support and depressive symptoms, with a significant indirect effect of −0.033 (95% *CI*: −0.056 to −0.016), accounting for 22.3% of the total effect.

**Conclusions:**

This study revealed significant heterogeneity in illness uncertainty among patients with gynecologic and breast cancers, with class membership associated with multiple factors including caregiver type and time since diagnosis. Illness uncertainty appeared to mediate the relationship between social support and depressive symptoms. These findings may inform the development of stratified psychosocial interventions tailored to distinct uncertainty profiles, though further longitudinal research is needed to establish causal relationships.

## Introduction

Gynecologic and breast cancers represent a predominant health concern for women globally and are associated with a substantial disease burden [1,2]. According to the GLOBOCAN 2022 estimates released by the International Agency for Research on Cancer (IARC), breast cancer was the most frequently diagnosed cancer in women globally, representing 23.8% of all new female cancer cases [3]. Gynecologic malignancies, such as cervical, ovarian, and endometrial cancers, also exhibited high incidence rates, collectively accounting for approximately 1,470,000 new cases worldwide in 2022 [3]. Beyond the physical toll, these cancers impose profound cognitive and emotional challenges, as patients must navigate complex diagnostic processes, intensive treatment regimens, and uncertainties about long-term prognosis [4]. In China, disparities in healthcare resources and an underdeveloped supportive care infrastructure often leave patients with insufficient information and psychological support [5], further exacerbating their psychological distress.

Illness uncertainty is a common psychological challenge among patients with gynecologic and breast cancers [6,7]. According to Mishel’s Uncertainty in Illness Theory, illness uncertainty refers to cognitive distress arising from the inability to interpret illness-related events, assign meaning to symptoms, or predict outcomes [8,9]. In the cancer context, patients frequently encounter multiple sources of uncertainty—ambiguity regarding diagnosis and prognosis, complexity of treatment information, lack of clear guidance for daily management, and unpredictability of disease trajectory [10]. Relevant studies indicate that a substantial proportion of cancer patients experience moderate-to-high levels of illness uncertainty [11], which significantly impairs their treatment coping [12], quality of life [13], and long-term adaptation [14]. In China, limited supportive care resources may further intensify these feelings [5], contributing to anxiety, depression, and other psychological issues. Therefore, systematic identification of high-risk subgroups and elucidation of the underlying mechanisms have become imperative in gynecologic oncology nursing [15].

A growing body of evidence has established important relationships among social support, illness uncertainty, and depressive symptoms in cancer patients. According to the social support buffering model, social support serves as a protective resource that mitigates the adverse effects of stress on psychological well-being [16]. Empirical studies have consistently confirmed that higher levels of perceived social support are associated with lower depression severity in cancer populations [17]. Concurrently, social support is negatively associated with illness uncertainty, with recent meta-analytic evidence demonstrating a moderate negative correlation between these two constructs [18], suggesting that support may reduce uncertainty by providing information, emotional reassurance, and practical assistance. Illness uncertainty itself, in turn, confers significant risk for depressive symptoms [11,13], as persistent cognitive ambiguity depletes psychological resources and fosters helplessness. The mediating role of uncertainty between social support and depression has been observed in other cancer populations [19], yet this pathway remains inadequately examined in patients with gynecologic and breast cancers.

In recent years, latent profile analysis (LPA) has been extensively applied to study psychological characteristics among breast cancer patients [20,21], successfully identifying distinct subgroups with varied adaptation patterns [22]. These person-centered approaches have revealed that patients do not experience psychological distress uniformly, and that sociodemographic and clinical factors may correlate with subgroup membership [23]. However, such research on illness uncertainty remains relatively scarce in the field of gynecological malignancies. Meanwhile, structural equation modeling (SEM) is widely used to examine complex pathways among variables [12,24], but few studies have employed both LPA and SEM to address heterogeneity and mechanistic relationships concurrently. Within the Chinese context, researchers have begun to recognize the importance of illness uncertainty, yet most existing studies remain confined to descriptive analyses and exploration of influencing factors [25,26]. Thus, there is a need to move beyond homogeneous assumptions and to clarify how social support, uncertainty, and depression interact in this population.

To address these issues, the present study employs two complementary analytical approaches. First, latent profile analysis is used to identify potential subgroups of illness uncertainty among patients with gynecologic and breast cancers and to examine demographic and clinical factors associated with subgroup membership. Second, structural equation modeling is applied to test the hypothesized mediating role of illness uncertainty in the relationship between social support and depressive symptoms. These two methods are applied separately but provide complementary perspectives: LPA captures the heterogeneity of uncertainty experiences, while SEM elucidates the pathways linking support, uncertainty, and emotional outcomes. Grounded in Mishel’s theory [8] and the social support buffering model [16], we hypothesize that social support not only directly reduces depressive symptoms but also indirectly alleviates depression by decreasing illness uncertainty. The theoretical rationale is that adequate support can reduce cognitive ambiguity and enhance patients’ confidence in managing their illness, thereby attenuating uncertainty and its negative emotional consequences [18,27].

The objectives of this study are threefold: to describe the current status of illness uncertainty among patients with gynecologic and breast cancers, to identify latent heterogeneous classes and examine their associated factors, and to empirically test the mediating role of illness uncertainty between social support and depressive symptoms using structural equation modeling. By applying these complementary analytical approaches separately, we hope to gain a more nuanced understanding of the psychological adaptation process in this population, which may offer preliminary evidence for developing stratified psychosocial interventions to improve patients’ quality of life.

## Methods

### 1. Study design

A single-center, cross-sectional study was conducted at a tertiary hospital in Beijing from December 2024 to June 2025. Consecutive eligible patients were enrolled through convenience sampling. This study was performed in line with the principles of the Declaration of Helsinki. The protocol was approved by the Institutional Review Board of the hospital (2024PHB227-001), and written informed consent was obtained from all participants prior to enrollment.

### 2. Participants

The study included patients who were diagnosed with gynecologic or breast malignancies. These patients were all receiving treatment at the participating tertiary hospital. Inclusion criteria comprised: histopathologically confirmed primary gynecologic or breast cancer; age ≧ 18 years; sufficient literacy or communication skills to complete questionnaires independently or with minimal assistance; informed consent and voluntary participation. Exclusion criteria comprised: end-stage disease, hospice care, or critical conditions preventing study participation; experience of major stressful events or psychological trauma within the past three months, e.g., bereavement, major family changes, or divorce; current involvement in other interventional clinical trials.

### 3. Sample Size

Sample size estimation integrated requirements for cross-sectional study, LPA, and SEM. First, The sample size formula for cross-sectional studies, $n=\frac{Z_{\text{1}-\alpha /\text{2}}^{\text{2}}p(\text{1}-p)}{d^{\text{2}}}$ [28], Z_1−*α*/2_ set at 1.96 (*α* = 0.05), *p* set at 0.6 according to previous studies [11,29], and a margin of error *d* of 0.05, yielding a sample size of 369. Second, for latent profile analysis, a sample size greater than 300 is generally recommended to ensure the stability and reliability of class identification [30,31]. Third, regarding structural equation modeling, Kline [32] suggests that the sample size for SEM should be at least 200, or 5–10 times the number of free parameters in the model. In summary, a total of 428 patients with gynecological and breast cancers were recruited, with 413 ultimately included, resulting in a response rate of 96.5%.

### 4. Data collection

Data collection was conducted from December 2024 to June 2025 in private, quiet rooms at the outpatient clinic and inpatient wards of a tertiary hospital in Beijing. Eligible patients were identified by trained research nurses through daily screening of medical records and clinician referrals. Potential participants were approached consecutively during their clinic visits or hospitalization, and those who met the inclusion criteria were provided with a detailed verbal and written explanation of the study purpose, procedures, and confidentiality protections. After providing written informed consent, participants completed the questionnaire battery in a self-administered format. Research staff remained available throughout to clarify any questions and to assist those with limited literacy in reading the items without biasing responses. Each participant took approximately 10–20 minutes to complete the questionnaires. Upon completion, all questionnaires were immediately reviewed for completeness by the research staff to minimize missing data.

### 5. Measures

#### Sociodemographic and clinical information.

A self-administered questionnaire designed by the research team was used to collect sociodemographic and clinical characteristics. Based on prior literature documenting factors associated with illness uncertainty in cancer populations [5,29], the following variables were included as potential correlates of latent class membership: age (categorized as 19–30, 31–50, 51–70, and > 70 years), ethnicity (Han vs. ethnic minority), educational level (primary or below, junior high school, high school or vocational school, bachelor’s degree, master’s or above), place of residence (rural area, town, city, other), occupation (mental work, manual work, student, other, retired), monthly household income in CNY (< 2000, 2000–5000, 5001–10000, 10001–20000, > 20000), marital status (unmarried, married, divorced/separated, widowed), having children (yes/no), having religion (yes/no), primary caregiver (spouse, parents, sons/daughters, siblings, professional caregiver, other), cancer type (gynecologic cancer vs. breast cancer), history of smoking (yes/no), history of alcohol consumption (yes/no), and time since diagnosis (just diagnosed, ≤ 1 month, ≤ 3 months, ≤ 6 months). These variables were selected based on their associations with illness uncertainty, psychological adaptation, and social support in cancer populations [13,18].

#### Mishel Uncertainty in Illness Scale (MUIS).

The MUIS, originally developed by Mishel in 1981 [33], was used in its validated Chinese version [34]. The scale comprises 33 items across four subscales: Uncertainty with 13 items, Complexity with 7 items, Information Lack with 7 items, and Unpredictability with 5 items. Each item is rated on a 5-point Likert scale ranging from 1 to 5. Item 15 is not included in the total score; total scores therefore range from 32 to 160, with higher scores indicating higher levels of illness uncertainty. The subscale score ranges are: Uncertainty, 13–65; Complexity, 7–35; Information Lack, 7–35; and Unpredictability, 5–25. Reliability analyses demonstrated adequate internal consistency for the total scale with a Cronbach‘s *α* of 0.926. The subscale reliabilities were as follows: Uncertainty (*α* = 0.910), Complexity (*α* = 0.843), Information Lack (*α* = 0.776), and Unpredictability (*α* = 0.832).

#### Patient Health Questionnaire-9 (PHQ-9).

The PHQ-9, developed by Kroenke et al. in 2001 [35], was used to screen for depressive symptoms. This study referred to the Chinese version adapted by Bian et al [36]. It consists of 9 items, each scored from 0 to 3, yielding a total score between 0 and 27. Interpretation of scores: 0–4 = no depression, 5–9 = mild depression, 10–14 = moderate depression, 15–19 = moderately severe depression, and 20–27 = severe depression. The Cronbach’s *α* in this study was 0.877.

#### Generalized Anxiety Disorder-7 (GAD-7).

The GAD-7, developed by Spitzer et al. in 2006 [37], was employed to assess anxiety symptoms. This study used the version translated by He Xiaoyan et al [38]. It contains 7 items scored from 0 to 3, with total scores ranging from 0 to 21. Cut-off scores are: 0–4 = no anxiety, 5–9 = mild anxiety, 10–14 = moderate anxiety, and 15–21 = severe anxiety. The scale demonstrated a Cronbach’s *α* of 0.934 in this study.

#### Social Support Rating Scale (SSRS).

The SSRS was originally developed by Xiao Shuiyuan in 1986 [39]. The 10-item instrument comprises three dimensions: subjective support (4 items), objective support (3 items), and utilization of support (3 items). Items are scored differently based on response options, with total scores ranging from 12 to 66. Higher scores indicate higher levels of social support. The Cronbach’s *α* was 0.784.

### 6. Statistical analysis

Statistical analyses were conducted using SPSS 26.0, Mplus 8.3, and AMOS 29.0. Categorical variables were reported as frequency (%) and compared by *χ*² or *Fisher’s* exact test. Continuous variables were described using mean ± *SD* or median (*IQR*) and analyzed by *ANOVA* or *Kruskal–Wallis* test. Significant univariate predictors (*P* < 0.05) were included in a multivariate logistic regression to identify factors associated with latent class membership.

Latent profile analysis was performed using Mplus 8.3 with the individual item scores of the MUIS as continuous indicators. The MLR estimator was used for model estimation to provide standard errors and fit statistics robust to non-normality of the indicators. To avoid convergence to local maxima, we specified 5,000 random sets of starting values for each model, with the 500 best values retained for final optimization [40]. The optimal number of latent profiles was determined through a comprehensive evaluation of multiple statistical criteria and theoretical considerations. The following information-theoretic fit indices were examined: Akaike Information Criterion (AIC), Bayesian Information Criterion (BIC), and sample-size adjusted BIC (aBIC), with lower values indicating better fit. The Lo-Mendell-Rubin adjusted likelihood ratio test (LMR) and the bootstrap likelihood ratio test (BLRT) were used to compare models with k versus k − 1 classes, with a significant *P* value (*P* < 0.05) indicating that the model with k classes fits significantly better [41]. Classification quality was assessed using entropy, with values greater than 0.80 indicating acceptable classification accuracy [42]. The final model selection was based on an integrated evaluation of all fit indices, entropy values, likelihood ratio test results, and the theoretical interpretability and parsimony of each solution.

Structural equation modeling was conducted using AMOS 29.0 to examine the direct and indirect effects among social support, illness uncertainty, and depressive symptoms. In the hypothesized model, social support and depressive symptoms were specified as observed variables, while illness uncertainty was specified as a latent variable reflected by its observed indicators. The model was estimated using maximum likelihood estimation. Model fit was evaluated using the following indices with their corresponding acceptable thresholds: the comparative fit index (CFI) and Tucker-Lewis index (TLI) with values greater than 0.90 indicating acceptable fit and greater than 0.95 indicating good fit; the root mean square error of approximation (RMSEA) with values less than 0.08 indicating acceptable fit and less than 0.05 indicating good fit; and the ratio of chi-square to degrees of freedom (*χ*²/*df*) with values less than 3 indicating acceptable fit [43]. The significance of indirect effects was tested using bootstrap resampling with 5,000 bootstrap samples and 95% bias-corrected confidence intervals; an indirect effect was considered statistically significant if the 95% confidence interval did not contain zero [44].

## Results

### 1. Sociodemographic characteristics and illness uncertainty scores of the study participants

Among the 413 enrolled patients with gynecological or breast malignancies, the age range was 19–85 years, with the majority (48.7%) being between 51 and 70 years old. The cohort was predominantly Han Chinese (93.9%). Regarding educational attainment, 35.4% had completed high school or vocational college, and 24.2% held a bachelor’s degree. Most patients were married (85.2%) and had children (86.4%). The most common monthly household income range was 5,001–10,000 RMB (27.1%). Detailed characteristics are presented in Table 1.

**Table 1:** Factors associated with illness uncertainty classes in gynecologic and breast cancer patients.

Variable	Total (n = 413)	Class 1 (n = 33)	Class 2 (n = 155)	Class 3 (n = 225)	*χ* ^*2*^ */F*	*P*
**Age (year)**					3.910	0.683
19–30	21	1 (4.8)	8 (38.1)	12 (57.1)		
31–50	169	10 (5.9)	63 (37.3)	96 (56.8)		
51–70	201	19 (9.5)	78 (38.8)	104 (51.7)		
> 70	22	3 (13.6)	6 (27.3)	13 (59.1)		
**Ethnicity**					**6.847**	**0.026**
Han	388	33 (8.5)	150 (38.7)	205 (52.8)		
Ethnic minority	25	0 (0.0)	5 (20.0)	20 (80.0)		
**Education level**					**15.220**	**0.044**
Primary or below	49	3 (6.1)	16 (32.7)	30 (61.2)		
Junior high school	93	10 (10.8)	27 (29.0)	56 (60.2)		
High school or vocational school	146	16 (11.0)	54 (37.0)	76 (52.1)		
Bachelor’s degree	100	2 (2.0)	45 (45.0)	53 (53.0)		
Master’s or above	25	2 (8.0)	13 (52.0)	10 (40.0)		
**Residence**					**11.684**	**0.052**
Rural area	93	6 (6.5)	38 (40.9)	49 (52.7)		
Town	105	15 (14.3)	34 (39.4)	56 (53.3)		
City	212	11 (5.2)	83 (39.2)	118 (55.7)		
Other	3	1 (33.3)	0 (0.0)	2 (66.7)		
**Monthly Household income (CNY)**					5.587	0.695
< 2000	79	5 (6.3)	28 (35.4)	46 (58.2)		
2000 ~ 5000	108	14 (13.0)	41 (38.0)	53 (49.1)		
5001 ~ 10000	112	8 (7.1)	40 (35.7)	64 (57.1)		
10001 ~ 20000	70	4 (5.7)	27 (38.6)	39 (55.7)		
> 20000	44	2 (4.5)	19 (43.2)	23 (52.3)		
**Marital status**					5.007	0.509
Unmarried	23	0 (0.0)	7 (30.4)	16 (69.6)		
Married	352	29 (8.2)	136 (38.6)	187(53.1)		
Dissociation	20	2 (10.0)	5 (25.0)	13 (65.0)		
Widowed	18	2 (11.1)	7 (38.9)	9 (50.0)		
**Has children**					1.862	0.409
Yes	357	31 (8.7)	135 (37.8)	191 (53.5)		
No	56	2 (3.6)	20 (35.7)	34 (60.7)		
**Occupation**					8.615	0.349
Mental work	86	2 (2.3)	37 (43.0)	47 (54.7)		
Manual work	58	4 (6.9)	22 (37.9)	32 (55.2)		
Student	4	0 (0.0)	2 (50.0)	2 (50.0)		
Other	138	13 (9.4)	45 (32.6)	80 (58.0)		
Retired	127	14 (11.0)	49 (38.6)	64 (50.4)		
**Has religion**					3.888	0.121
Yes	21	0 (0.0)	12 (57.1)	9 (42.9)		
No	392	33 (8.4)	143 (36.5)	216 (55.1)		
**Primary caregiver**					**18.268**	**0.036**
Spouse	226	13 (5.8)	92 (42.7)	121 (53.5)		
Parents	31	1 (3.2)	9 (29.0)	21 (67.7)		
Sons and daughters	80	8 (10.0)	22 (27.5)	50 (62.5)		
Siblings	17	1 (5.9)	7 (41.2)	9 (52.9)		
Professional caregiver	35	7 (20.0)	12 (34.3)	16 (45.7)		
Other	24	3 (12.5)	13 (54.2)	8 (33.3)		
**Cancer type**					**7.804**	**0.020**
Gynecologic cancer	212	24 (11.3)	82 (38.7)	106 (50.0)		
Breast cancer	201	9 (4.5)	73 (36.3)	119 (59.2)		
**History of smoking**					1.921	0.348
Yes	15	0 (0.0)	4 (26.7)	11 (73.3)		
No	398	33 (8.3)	151 (35.9)	214 (53.8)		
**History of alcohol consumption**					2.292	0.356
Yes	27	33 (8.5)	144 (37.3)	209 (54.1)		
No	386	0 (0.0)	11 (40.7)	16 (59.3)		
**Time since diagnosis**					**14.815**	**0.021**
Just diagnosed	72	6 (8.3)	16 (22.2)	50(69.4)		
≤ 1 month	115	10 (8.7)	50 (43.5)	55(47.8)		
≤ 3 months	78	10 (12.8)	27 (35.6)	41(52.6)		
≤ 6 months	148	7 (4.7)	62 (41.9)	79 (53.4)		
**Depression (Mean±SD)**		1.27 ± 2.06	4.12 ± 4.47	6.01 ± 5.28	**17.456**	**<0.001**
**Social support (Mean±SD)**		46.33 ± 7.75	44.01 ± 7.73	40.81 ± 7.49	**12.807**	**<0.001**
**Anxiety (Mean±SD)**		0.94 ± 1.95	2.97 ± 4.22	4.80 ± 4.91	**14.895**	**<0.001**

Abbreviations: CNY, Chinese Yuan, 1 USD ≈ 7.1 CNY at the time of data collection.

Notes: Data are presented as n (%) unless otherwise specified. Bolded values in the *χ*²/*F* and *P* value columns indicate statistical significance.

The total mean score for illness uncertainty was 94.32 ± 18.21. The dimension scores, in descending order, were as follows: Complexity (25.61 ± 4.81, mean item score 3.65 ± 0.69), Unpredictability (15.41 ± 3.33, mean item score 3.08 ± 0.67), Uncertainty (36.54 ± 9.93, mean item score 2.81 ± 0.76), and Information Lack (16.77 ± 4.83, mean item score 2.40 ± 0.69).

### 2. Latent profile analysis of illness uncertainty and class characteristics

LPA was conducted using the raw scores of the 33 items from MIUS as manifest variables, preserving the clinical interpretability of the original Likert scale. Models ranging from 1 to 5 classes were fitted and compared based on model fit indices (Table 2). The three-class model demonstrated the optimal fit: Entropy was 0.941, indicating high classification accuracy. LMR was significant (*P* = 0.043), and BLRT was also significant (*P* < 0.001), confirming that the three-class model provided a significantly better fit than the two-class model. Consequently, the three-class model was selected as the final solution.

**Table 2:** Fit indices for latent profile models of illness uncertainty (n = 413).

Model	LL	AIC	BIC	aBIC	Entropy	LMR (*P)*	BLRT (*P)*	Latent Class Probability
1	−19792.044	39716.088	39981.635	39772.202				1.000
2	−18561.557	37323.114	37725.459	37408.137	0.958	0.002	<0.001	0.668/0.332
**3**	**−17890.567**	**36049.133**	**36588.275**	**36163.063**	**0.941**	**0.043**	**<0.001**	**0.080/0.544/0.376**
4	−17639.998	35615.996	36291.935	35758.834	0.905	0.766	<0.001	0.073/0.380/0.249/0.298
5	−17442.864	35289.729	36102.465	35461.474	0.911	0.705	<0.001	0.070/0.225/0.153/0.397/0.155

Notes: The selected 3-class model is presented in bold. AIC, BIC, and aBIC are information-theoretic criteria where lower values indicate better model fit. Entropy ranges from 0 to 1, with values closer to 1 indicating clearer classification. A significant LMR test (*P* < 0.05) suggests that the model with k classes fits significantly better than a model with k-1 classes.

**Fig 1:**
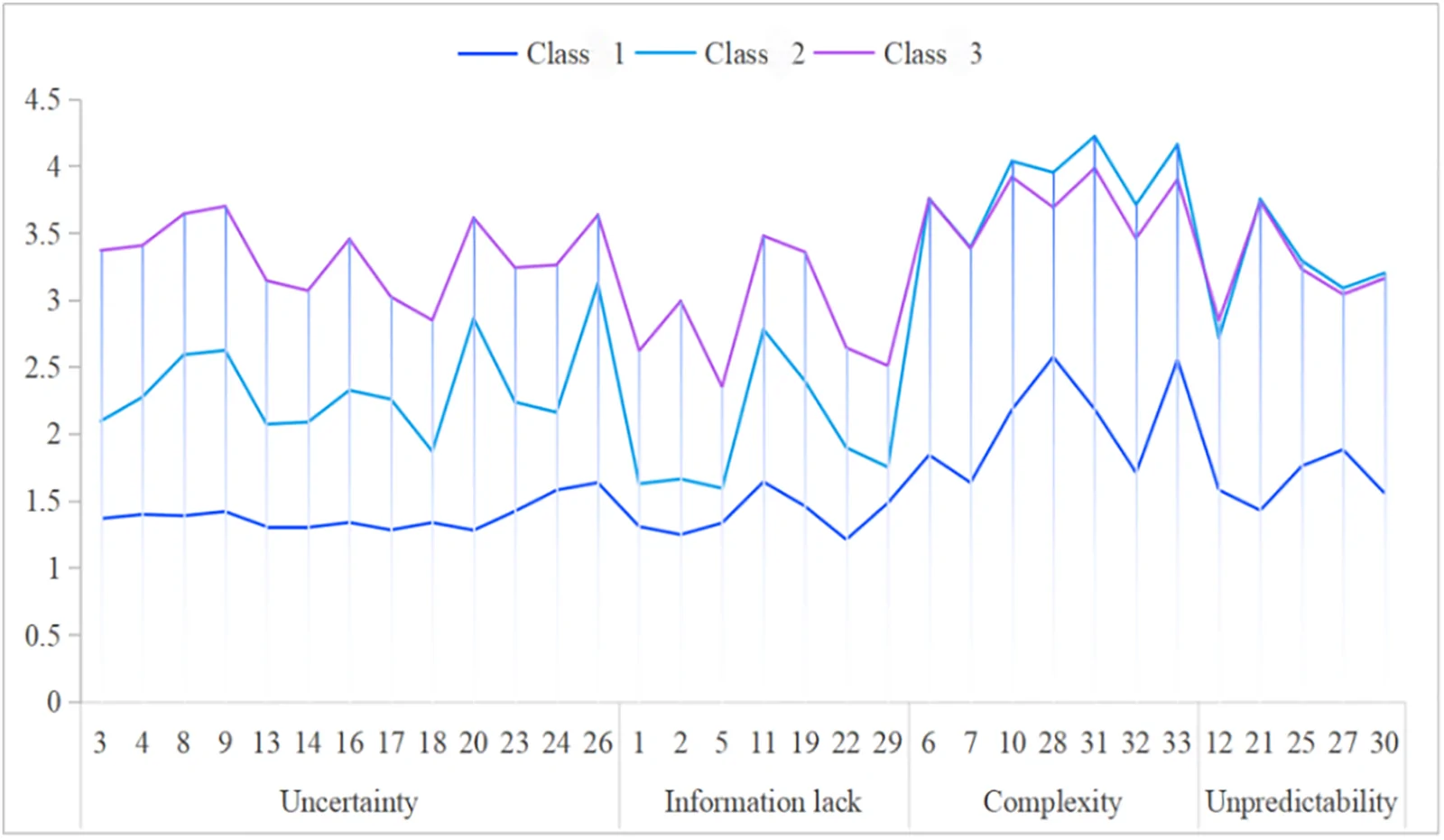
Distribution of characteristics of three potential categories of illness uncertainty. Notes: Three distinct latent classes were identified through latent profile analysis. Class 1 (8.0%): Characterized by the lowest scores across all MUIS dimensions. Class 2 (37.6%): Exhibited moderately elevated scores across all subscales, with the highest relative score on the Complexity subscale (mean = 3.73), which was comparable to that of Class 3 (mean = 3.89). Class 3 (54.4%): Dominated by high scores on the Uncertainty, Information Lack, and Unpredictability subscales, with Complexity also elevated (mean = 3.89) (class-specific subscale means are presented in S1 Table).

The classes were named based on their distinct illness uncertainty profiles (class-specific subscale means are presented in S1 Table). Class 1 (Low Uncertainty, Psychologically Adapted Group, n = 33, 8.0%) exhibited the lowest scores across all subscales. Class 2 (Moderate Uncertainty, Complexity Distress Group, n = 155, 37.6%) showed moderately elevated scores across all subscales, with a Complexity score (3.73 ± 0.83) comparable to that of Class 3 (3.89 ± 0.83). Class 3 (High Uncertainty, Cognitive Ambiguity-Dominant Group, n = 225, 54.4%) was characterized by the highest scores on Uncertainty, Information Lack, and Unpredictability. Class scores are detailed in Fig 1.

### 3. Univariate analysis of illness uncertainty classes in patients with gynecological and breast malignancies

Univariate analysis results (Table 1) revealed statistically significant differences (*P* < 0.05) in illness uncertainty class membership based on ethnicity, educational level, primary caregiver, cancer type, and time since diagnosis.

### 4. Multivariate analysis of illness uncertainty classes in patients with gynecological and breast malignancies

Variables identified as statistically significant in the univariate analysis were assigned values and included in a multivariate model. Using the largest class, the “High Uncertainty - Cognitive Ambiguity-Dominant Group (Class 3)”, as the reference group, a multinomial logistic regression analysis was performed (Table 3).

**Table 3:** Determinants of illness uncertainty class membership: a multinomial logistic regression.

Class	Variable	*B*	*SE*	*Wald χ* ^*2*^	*P*	*OR*	*95% CI*	
**Class 1** **vs.** **Class 3**	**Disease diagnosis**							
	Breast cancer	1.872	0.593	9.966	0.002	6.501	2.034	20.785
	**Education level**							
	Junior high school	2.281	1.116	4.181	0.041	9.787	1.099	87.130
	Bachelor’s degree	2.794	1.185	5.559	0.018	16.352	1.602	166.865
	**Primary caregiver**							
	Spouse	2.208	0.965	5.233	0.022	9.099	1.372	60.352
	**Time since diagnosis**							
	Just diagnosed	−1.246	0.657	3.590	0.058	0.288	0.079	1.044
	≤ 3 months	−1.356	0.686	3.914	0.048	0.258	0.067	0.987
	**Depression**	−0.300	0.112	7.217	0.007	0.741	0.595	0.922
**Class 2** **vs.** **Class 3**	**Ethnicity**							
	Han	1.337	0.563	5.635	0.018	3.808	1.262	11.484
	**Education level**							
	Primary or below	1.955	0.704	7.704	0.006	7.066	1.776	28.103
	Junior high school	1.708	0.596	8.204	0.004	5.517	1.715	17.751
	High school or vocational school	1.113	0.537	4.305	0.038	3.045	1.064	8.716
	**Primary caregiver**							
	Spouse	1.030	0.521	3.906	0.048	2.800	1.009	7.772
	Parents	1.859	0.681	7.447	0.006	6.417	1.688	24.387
	Sons and daughters	1.230	0.586	4.404	0.036	3.423	1.085	10.801
	**Time since diagnosis**							
	Just diagnosed	0.899	0.401	5.037	0.025	2.457	1.121	5.387
	**Social support**	0.044	0.017	7.144	0.008	1.045	1.012	1.079

Abbreviations: Class 1, Low Uncertainty Group; Class 2, Moderate Uncertainty Group; Class 3, High Uncertainty Group (used as the reference category in this model).

Notes: The analysis was adjusted for all variables presented in the table. Only statistically significant predictors from the multivariate model are shown here for clarity.

For Class 1 vs. Class 3, significant protective factors included a diagnosis of pelvic tumor, an educational level of junior high school or bachelor’s degree, having a spouse as the primary caregiver, and a lower PHQ-9 depression score (*OR* = 0.741, *P* = 0.007). For Class 2 vs. Class 3, significant predictors were Han ethnicity, lower educational attainment, having a spouse (*OR* = 2.800, *P* = 0.048), parent (*OR* = 6.417, *P* = 0.006), or child (*OR* = 3.423, *P* = 0.036) as the primary caregiver, a shorter time since diagnosis (*OR* = 2.457, *P* = 0.025), and a higher level of social support (*OR* = 1.045, *P* = 0.008). The results indicate that educational level, caregiver type, time since diagnosis, depressive symptoms, and social support are significant predictors distinguishing the different illness uncertainty classes.

### 5. Correlations and structural equation modeling analysis of illness uncertainty and psychosocial factors

**Fig 2:**
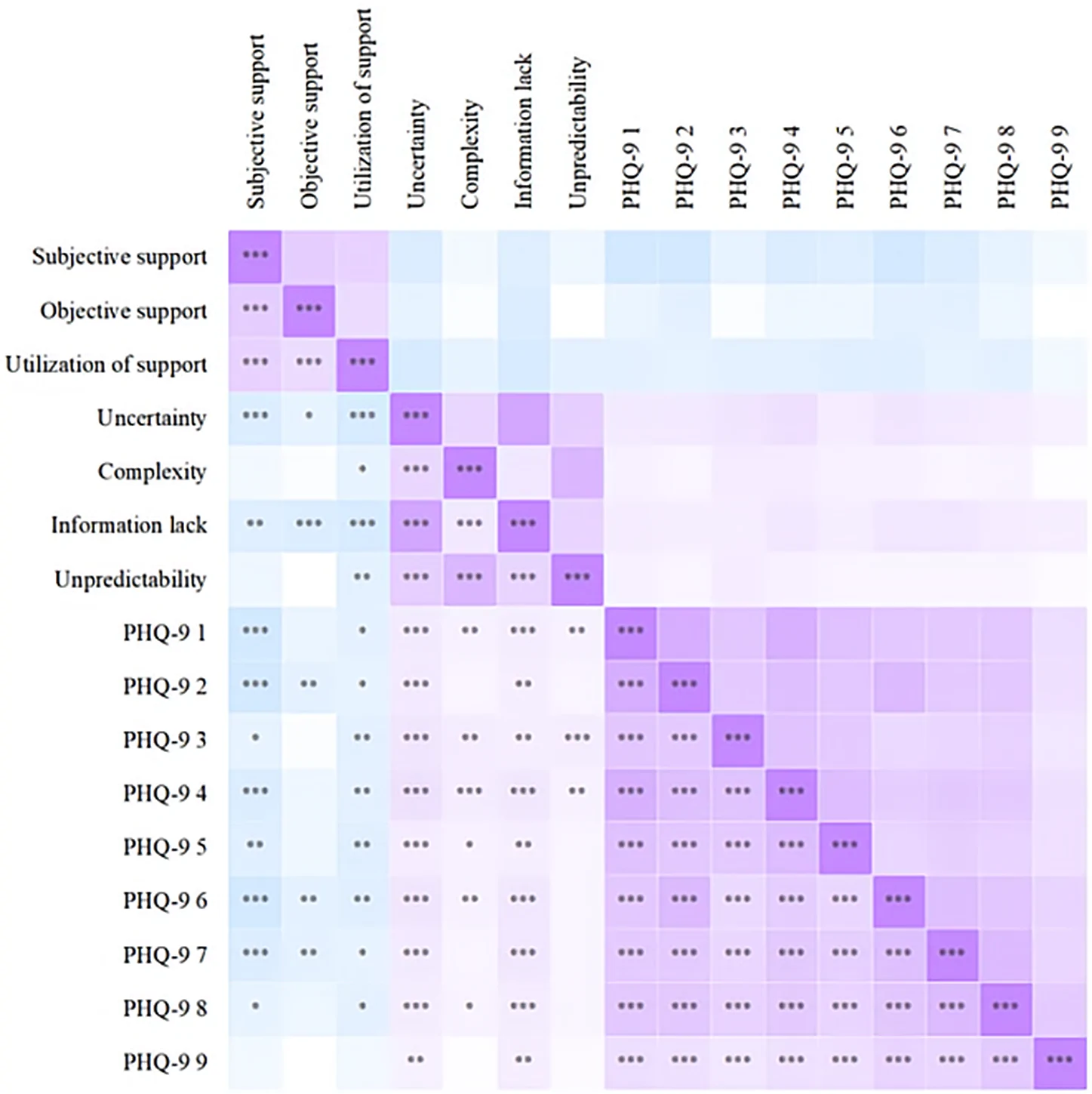
Bivariate correlations among illness uncertainty, depressive symptoms, and social support. Notes: The heatmap illustrates Pearson correlation coefficients between the total scores of the Mishel Uncertainty in Illness Scale (MUIS), the Patient Health Questionnaire-9 (PHQ-9), and the Social Support Rating Scale (SSRS).

A correlation heatmap illustrating the relationships between MUIS, PHQ-9, and SSRS scores is shown in Fig 2. Confirmatory factor analyses were conducted to evaluate the measurement models for SSRS and MUIS. For SSRS, the three-factor model showed acceptable fit, with standardized factor loadings of 0.72, 0.68, and 0.65 for the three dimensions (*P* < 0.001). Composite reliability (*CR*) was 0.78, and average variance extracted (*AVE*) was 0.54. For MUIS, the four-factor model also demonstrated acceptable fit, with standardized factor loadings of 0.81, 0.74, 0.76, and 0.69 for the four subscales (*P* < 0.001). *CR* was 0.86, and *AVE* was 0.61. These results support the adequacy of the measurement models. PHQ-9 was treated as an observed variable (total score) in the structural model and therefore was not subjected to CFA. The factor loadings and reliability estimates are presented in S1 Table.

**Fig 3:**
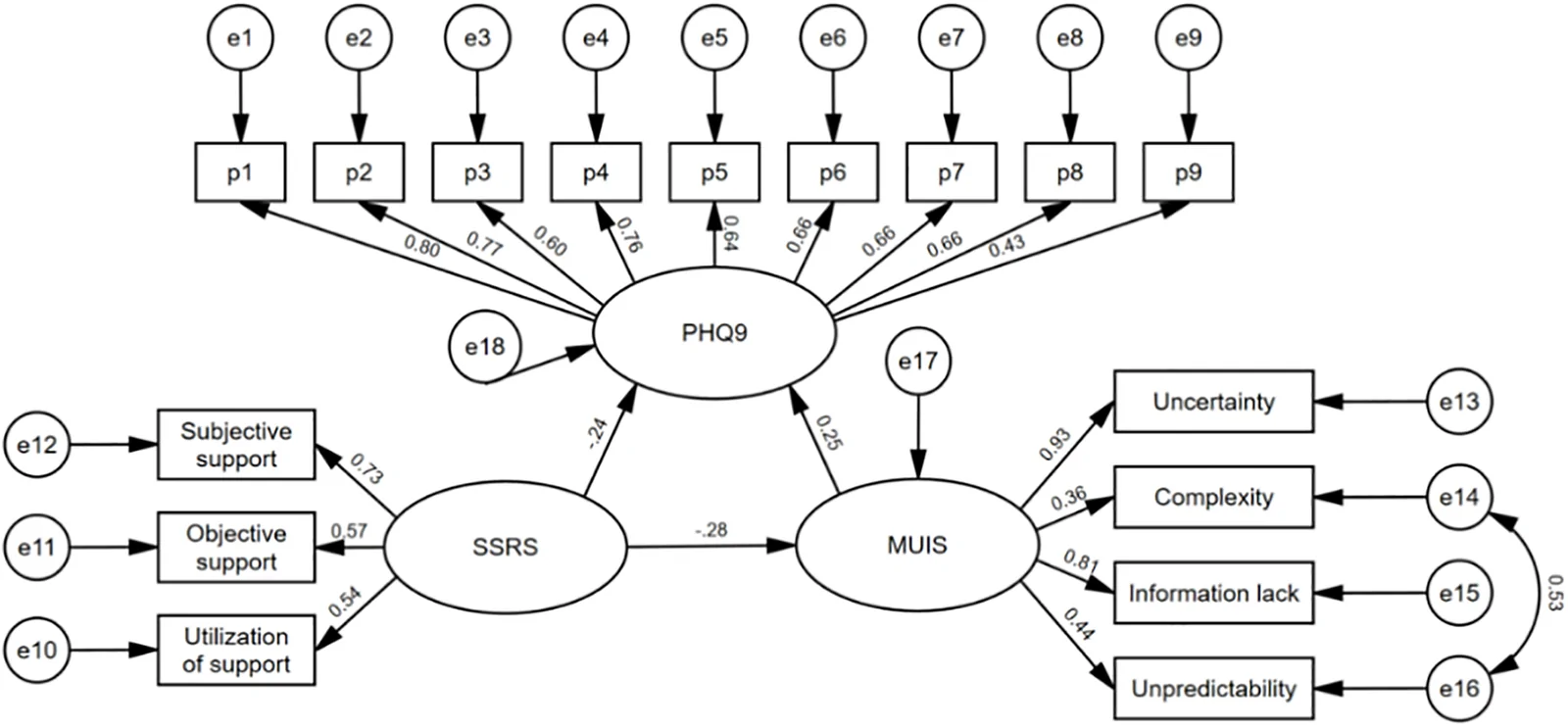
Structural equation model testing the mediating role of illness uncertainty (revised). Notes: The model depicts the relationships between social support, illness uncertainty, and depressive symptoms. Standardized path coefficients are shown. Model fit indices confirmed a good fit: *χ²*/df = 2.769, RMSEA = 0.066, IFI = 0.928, CFI = 0.927, TLI = 0.912. *P* < 0.001. Based on modification indices, one covariance between the residual terms of the Ambiguity and Information Lack subscales was added, as these two dimensions are theoretically linked within Mishel’s framework. No other post-hoc modifications were made.

SEM was employed to examine the pathway relationships among the variables (Fig 3). The modified model demonstrated a good fit, with all indices meeting acceptable standards. Path analysis indicated that social support was negatively associated with illness uncertainty (*β* = −0.28, *P* < 0.001), and illness uncertainty was positively associated with depressive symptoms (*β* = 0.25, *P* < 0.001). To formally test the mediating role of illness uncertainty, we conducted bootstrapped mediation analysis using AMOS 29.0 with 5,000 bootstrap resamples. As shown in Table 4, the indirect effect of social support on depressive symptoms through illness uncertainty was −0.033 (95% *CI*: −0.056 to −0.016). Since the 95% bias-corrected bootstrap confidence interval did not contain zero, the indirect effect was statistically significant. The direct effect remained significant (*c*‘ = −0.114, *P* < 0.001), indicating partial mediation. The indirect effect accounted for 22.3% of the total effect.

**Table 4:** Effects of social support on depressive symptoms via illness uncertainty.

Effect	Estimate	*S.E.*	Bias-corrected 95% *CI*	*P*
Total effect (SSRS → PHQ-9)	−0.147	0.031	[−0.207, −0.087]	<0.001
Direct effect (SSRS → PHQ-9)	−0.114	0.030	[−0.174, −0.055]	<0.001
Indirect effect (SSRS → MUIS → PHQ-9)	−0.033	0.010	[−0.056, −0.016]	<0.001

Notes: *S.E*. = bootstrap standard error; *CI* = confidence interval. Bootstrap resampling with 5,000 samples was used to compute bias-corrected confidence intervals. An indirect effect is considered statistically significant if the 95% confidence interval does not contain zero. The indirect effect accounted for 22.3% of the total effect.

## Discussion

### 1. Heterogeneity in illness uncertainty among individuals with gynecologic and breast cancer

The study primarily consisted of middle-aged and older adults (51–70 years, 48.7%), with most patients in the early post-diagnosis phase. Patients generally experienced moderate-to-high levels of illness uncertainty, with treatment complexity perceived most strongly, followed by unpredictability of disease progression. This pattern aligns with Kang et al., who similarly reported a strong perception of unpredictability among cancer patients [45]. Crucially, LPA identified three distinct latent classes of illness uncertainty, suggesting significant heterogeneity within this population. This finding is consistent with person-centered research in gynecologic oncology. For instance, latent profile analyses of reproductive concerns among young women with cervical cancer and symptom clusters in gynecologic malignancies have similarly revealed distinct patient subgroups [46]. In breast cancer populations, latent profile analysis has likewise revealed heterogeneous subgroups of family adaptation [22].

The Low Uncertainty-Psychological Adaptation Group (8.0%), the smallest profile, exhibited the lowest scores across all uncertainty dimensions. They also showed mild anxiety and depression and the highest level of social support, may suggest greater psychological resilience. The Moderate Uncertainty-Complexity Distress Group (37.6%) was characterized by a notably elevated score on the complexity dimension (mean = 3.73 ± 0.83), comparable to that of the High Uncertainty Group (mean = 3.89 ± 0.83). The core stressor for this group likely stems from confusion and difficulty in managing complex treatment regimens and daily self-care demands [47]. The similarity in complexity scores between Class 2 and Class 3 suggests that complexity-related distress may represent a distinct dimension of psychological burden rather than being exclusive to those with the highest overall uncertainty. The High Uncertainty-Cognitive Ambiguity Dominant Group (54.4%) constituted the majority. Their defining feature was the heaviest burden on cognitive dimensions: ambiguity regarding diagnosis and prognosis, lack of information, and unpredictability of the future. This profound cognitive ambiguity was associated with the most severe anxiety, depressive symptoms, and the lowest social support, potentially creating a vicious cycle consistent with Mishel‘s Uncertainty in Illness Theory [33]. The predominance of this high-uncertainty group (54.4%) is higher than rates reported in long-term cancer survivors [48,49], suggesting that uncertainty may diminish over time as patients accumulate illness-related experiences and develop coping strategies.

### 2. Factors associated with illness uncertainty profiles

Multivariate logistic regression identified independent correlates of class membership. Higher social support (*OR* = 1.045, *P* = 0.008) and lower depression levels (*OR* = 0.741, *P* = 0.007) were significantly associated with membership in the low-uncertainty group. These findings align with a meta-analysis by Wu et al. [18], encompassing 41 studies involving 5,403 patients, which confirmed a moderate negative correlation between illness uncertainty and social support (*r* = −0.33). A shorter time since diagnosis (newly diagnosed: *OR* = 0.288; within 3 months: *OR* = 0.258) was also associated with lower uncertainty class membership, which contrasts with the findings of Shen et al. [11] in newly diagnosed cancer patients. We hypothesize that the initial diagnosis period may shift the cognitive focus from “what might be wrong” to “what to do now,” with clear treatment plans and concentrated support creating a temporary buffer. This interpretation is consistent with Kienzler et al. [10], who found that uncertainty is often most acute during periods of transition and information gaps. However, we acknowledge that time since diagnosis is closely related to treatment phase, which may influence uncertainty through different mechanisms; without controlling for treatment phase, this interpretation remains speculative. The “primary caregiver” variable was significantly associated with class membership. Patients with professional caregivers were more likely to belong to the low-uncertainty group compared to those cared for by family members, possibly reflecting that professional caregivers possess more structured knowledge about disease management and are better equipped to provide accurate information, thereby reducing cognitive ambiguity [50]. Future interventions should consider including caregiver assessment and training [51].

### 3. The pathway linking illness uncertainty, social support, and depressive symptoms

The SEM revealed a direct negative association between social support and illness uncertainty, suggesting that a robust support system may buffer illness-related uncertainty. Patients with high support may gain clearer information and effective coping strategies, reducing confusion and unpredictability [52]. Simultaneously, illness uncertainty showed a direct positive association with depressive symptoms, consistent with prior research [53]. Social support exhibited a direct negative association with depressive symptoms, corroborating findings from prior research, Among cervical cancer patients undergoing radiotherapy, anxiety and depression were found to be significantly correlated with illness uncertainty [54]. Furthermore, a cross-sectional study of female cancer survivors demonstrated that social support mediated the relationship between uncertainty in illness and quality of life [55], suggesting that social support may both indirectly alleviate depression by reducing uncertainty and serve as a direct protective factor. During model estimation, one post-hoc modification was made based on modification indices: a covariance between the residual terms of the Ambiguity and Information Lack subscales was added. This modification was theoretically grounded in Mishel‘s framework, as information deficiency is a primary contributor to cognitive ambiguity, and items from these two subscales share conceptual overlap in measuring patients’ perceived clarity regarding their illness [8,9]. The modification was minimal and did not alter any substantive structural paths. No other post-hoc modifications were made.

The mediation analysis supported the hypothesis that illness uncertainty partially mediates the relationship between social support and depressive symptoms. The indirect effect was −0.033 (95% *CI*: −0.056 to −0.016), accounting for 22.3% of the total effect. This finding aligns with Wang’s actor-partner interdependence mediation model in lung cancer patient-caregiver dyads, which demonstrated that illness uncertainty mediated the effect of perceived social support on anxiety and depression [19]. The consistency of this mediation pattern across different cancer populations suggests that the pathway from social support through illness uncertainty to depressive symptoms may represent a general mechanism of psychological adaptation in cancer, supporting Mishel‘s theoretical framework.

### 4. Study highlight and clinical implications

This study contributes to the limited body of research applying LPA and SEM in a complementary manner to the same patient population, and may offer preliminary insights into the heterogeneity of illness uncertainty and its psychosocial correlates. The identification of three distinct profiles provides a potential evidence base for precise psychological interventions. The High and Moderate uncertainty profiles together accounted for 92% of the sample, indicating the vast majority experience significant uncertainty, suggesting that uncertainty may be particularly acute during the active treatment phase and may diminish over time. The Moderate Uncertainty Group (37.6%) exhibited a complexity score comparable to that of the High Uncertainty Group, suggesting that complexity-related distress is shared across both groups. Interventions for this group might focus on managing complexity, such as providing structured treatment plans, medication guides, and symptom monitoring tools, and utilizing nurse navigation or case management to simplify processes and build confidence [56,57]. The SEM findings identified illness uncertainty as a potential core mediator between social support and depression, supporting a tripartite Cognitive-Support-Emotional intervention framework that warrants further evaluation. First, cognitive clarification: for Class 2 and Class 3 groups, provide individualized cognitive restructuring [58] and clear, culturally appropriate information to reduce ambiguity and complexity. Second, support optimization: implement caregiver education, family meetings, and peer support groups to enhance support quality [59,60]. Third, emotional regulation: incorporate mindfulness-based interventions or cognitive behavioral therapy to alleviate anxiety and depression [61] and break the uncertainty-emotional distress cycle.

## Limitations

Several limitations should be considered when interpreting the findings. First, the cross-sectional design precludes causal inference; although our mediation model is theoretically grounded, temporal precedence cannot be established, and reverse causation cannot be ruled out. Second, regarding the LPA approach, preliminary analyses using the four MUIS subscale scores as indicators resulted in unstable model estimation—entropy values fell below acceptable thresholds and BIC failed to converge across multiple class solutions. Consequently, we adopted an item-level approach using the 33 individual MUIS items to preserve the full granularity of patients’ response patterns and achieve a well-separated, interpretable three-class solution. While this approach has been employed in similar studies of illness perception and symptom patterns in cancer populations, it substantially increases the number of free parameters and may limit replicability; future studies with larger samples should validate the stability of these profiles. Third, the mediation model was estimated without adjusting for sociodemographic or clinical covariates. Including multiple categorical covariates (e.g., education, marital status, caregiver type) in a latent variable framework would require dummy coding, substantially increasing free parameters and potentially compromising estimation stability given the sample size. Similarly, anxiety (GAD-7) was excluded due to its high conceptual and empirical overlap with depression (PHQ-9), including both would introduce substantial multicollinearity and obscure the unique effects of each construct. This approach is consistent with previous mediation studies in oncology populations that prioritized theoretical parsimony [62,63]. Fourth, given the single-center design and the use of convenience sampling, caution is warranted when generalizing the findings to broader populations. Additionally, the sample size did not permit stratified analyses by cancer type, which would have been valuable for exploring disease-specific patterns. Future multicenter longitudinal studies with larger samples are needed to validate these findings across diverse populations and to examine the temporal dynamics of illness uncertainty and its impact on long-term psychological adjustment.

## Conclusion

This study applied latent profile analysis and structural equation modeling to examine the heterogeneity of illness uncertainty and its psychosocial correlates in patients with gynecologic and breast cancers. Three distinct profiles were identified—low uncertainty-psychologically adapted (8.0%), moderate uncertainty-complexity distress (37.6%), and high uncertainty-cognitive ambiguity (54.4%), suggesting that patients experience illness uncertainty in qualitatively different ways. Multivariate analyses indicated that caregiver type, time since diagnosis, social support, and depressive symptoms were associated with profile membership. Furthermore, mediation analysis suggested that illness uncertainty may partially mediate the relationship between social support and depressive symptoms (indirect effect = −0.033, 95% CI: −0.056 to −0.016), accounting for approximately 22% of the total effect. These findings point to the potential value of developing risk-stratified intervention strategies tailored to distinct uncertainty profiles, with integrated approaches addressing cognitive clarification, support enhancement, and emotional regulation. However, given the cross-sectional design, causal interpretations are not warranted; future longitudinal studies are needed to establish temporal relationships and evaluate the effectiveness of such targeted interventions.

## Supporting information

S1 Table Supplementary statistical tables providing additional analyses supporting the main findings of this study. (DOCX)

S2 Dataset Original dataset containing data from the 413 study participants. (XLSX)

S3 Questionnaire The complete survey questionnaire used in this study. (DOCX)
