# Supplementary material for: Illness uncertainty in individuals with gynecologic and breast cancer: A latent profile analysis and structural equation modeling
Source: PLoS One. 2026 Sep 18;21(9):e0358366. doi: 10.1371/journal.pone.0358366 (PMC13588340; doi:10.1371/journal.pone.0358366)
Supplement: Supplementary file 1 [file pone.0358366.s001.docx]

Table 5. Scores of the scale for uncertainty in patients with gynecological and breast tumors (score, *x̅*±s)

| Variable | Number of entries | Score | Average score perentry | Sort |
| --- | --- | --- | --- | --- |
| Uncertainty | 13 | 36.54±9.93 | 2.81±0.76 | 3 |
| Complexity | 7 | 25.61±4.81 | 3.65±0.69 | 1 |
| Information lack | 7 | 16.77±4.83 | 2.40±0.69 | 4 |
| Unpredictability | 5 | 15.41±3.33 | 3.08±0.67 | 2 |
| Total score of disease uncertainty scale | 33 | 94.32±18.21 | 2.95±0.57 |  |

Table 6. Class-specific means and standard deviations of MUIS subscales

| Subscale | Low Uncertainty Group (C1, n=33) | Moderate Uncertainty Group (C2, n=155) | High Uncertainty Group (C3, n=225) |
| --- | --- | --- | --- |
| Uncertainty | 1.36 ± 0.90 | 2.35 ± 0.90 | 3.26 ± 0.90 |
| Information Lack | 1.38 ± 0.91 | 1.96 ± 0.91 | 2.85 ± 0.91 |
| Complexity | 2.10 ± 0.83 | 3.73 ± 0.83 | 3.89 ± 0.83 |
| Unpredictability | 1.64 ± 0.85 | 3.21 ± 0.85 | 3.20 ± 0.85 |

Table 7. Variable assignment table of multiple logistic regression analysis

| Variable | Assignment |
| --- | --- |
| Nation | Han = 1; Ethnic minorities =2 |
| Degree of education | Primary school and below (1,0,0,0,0); Junior high school (0,1,0,0,0); Senior high school or college (0,0,1,0,0); Undergraduate (0,0,0,1,0); Graduate or above (0,0,0,0,1) |
| Domicile | Rural (1,0,0,0); Town (0,1,0,0); Urban (0,0,1,0); Other (0,0,0,1) |
| Key caregivers during illness | Spouse (1,0,0,0,0,0); Parents (1,0,0,0,0,0); Children (1,0,0,0,0,0); Brothers and sisters (1,0,0,0,0,0); Caregivers (1,0,0,0,0,0); Other (1,0,0,0,0,0) |
| Disease diagnosis | Gynecological tumor =1; Breast tumor =2 |
| Date of disease diagnosis | Newly diagnosed (1,0,0,0); 1 month (0,1,0,0); 3 months (0,0,1,0); 6 months (0,0,0,1) |
| PHQ-9 | Input of original values |
| SSRS | Input of original values |
| GAD-7 | Input of original values |

Table 8. Correlation analysis between MUIS, PHQ-9 and SSRS

|  | Subjective support | Objective support | Utilization of support | Uncertainty | Complexity | Informationlack | Unpredictability | PHQ-9 1 | PHQ-9 2 | PHQ-9 3 | PHQ-9 4 | PHQ-9 5 | PHQ-9 6 | PHQ-9 7 | PHQ-9 8 | PHQ-9 9 |
| --- | --- | --- | --- | --- | --- | --- | --- | --- | --- | --- | --- | --- | --- | --- | --- | --- |
| Subjective support | 1 |  |  |  |  |  |  |  |  |  |  |  |  |  |  |  |
| Objective support | 0.422* | 1 |  |  |  |  |  |  |  |  |  |  |  |  |  |  |
| Utilization of support | 0.383* | 0.310* | 1 |  |  |  |  |  |  |  |  |  |  |  |  |  |
| Uncertainty | -0.173* | -0.116* | -0.197* | 1 |  |  |  |  |  |  |  |  |  |  |  |  |
| Complexity | -0.048 | -0.002 | -0.100* | 0.358* | 1 |  |  |  |  |  |  |  |  |  |  |  |
| Information lack | -0.159* | -0.178* | -0.191* | 0.759* | 0.217* | 1 |  |  |  |  |  |  |  |  |  |  |
| Unpredictability | -0.067 | 0.004 | -0.130* | 0.406* | 0.605* | 0.375* | 1 |  |  |  |  |  |  |  |  |  |
| PHQ-9 1 | -0.204* | -0.093 | -0.122* | 0.196* | 0.134* | 0.170* | 0.132* | 1 |  |  |  |  |  |  |  |  |
| PHQ-9 2 | -0.214* | -0.135* | -0.120* | 0.184* | 0.084 | 0.149* | 0.083 | 0.680* | 1 |  |  |  |  |  |  |  |
| PHQ-9 3 | -0.115* | -0.012 | -0.132* | 0.231* | 0.161* | 0.145* | 0.166* | 0.487* | 0.446* | 1 |  |  |  |  |  |  |
| PHQ-9 4 | -0.170* | -0.071 | -0.137* | 0.276* | 0.180* | 0.220* | 0.136* | 0.665* | 0.549* | 0.514* | 1 |  |  |  |  |  |
| PHQ-9 5 | -0.143* | -0.068 | -0.151* | 0.168* | 0.122* | 0.143* | 0.095 | 0.533* | 0.462* | 0.445* | 0.565* | 1 |  |  |  |  |
| PHQ-9 6 | -0.21* | -0.137* | -0.140* | 0.224* | 0.132* | 0.214* | 0.096 | 0.468* | 0.581* | 0.339* | 0.432* | 0.354* | 1 |  |  |  |
| PHQ-9 7 | -0.169* | -0.134* | -0.1186* | 0.200* | 0.092 | 0.20979* | 0.087 | 0.448* | 0.458* | 0.353* | 0.469* | 0.402* | 0.554* | 1 |  |  |
| PHQ-9 8 | -0.110* | -0.073 | -0.122* | 0.170* | 0.119* | 0.166* | 0.092 | 0.476* | 0.466* | 0.389* | 0.452* | 0.368* | 0.494* | 0.590* | 1 |  |
| PHQ-9 9 | -0.0425 | 0.0127 | -0.043 | 0.135* | 0.033 | 0.146* | 0.054 | 0.294* | 0.277* | 0.214* | 0.275* | 0.275* | 0.374* | 0.341* | 0.454* | 1 |

Table 9. Factor loadings and reliability for measurement models

| Construct | Indicator | Standardized Loading | *CR* | *AVE* |
| --- | --- | --- | --- | --- |
| SSRS | Subjective Support | 0.72 | 0.78 | 0.54 |
|  | Objective Support | 0.68 |  |  |
|  | Utilization of Support | 0.65 |  |  |
| MUIS | Uncertainty | 0.81 | 0.86 | 0.61 |
|  | Complexity | 0.74 |  |  |
|  | Information Lack | 0.76 |  |  |
|  | Unpredictability | 0.69 |  |  |

Table 10. Mediation effect test of MUIS, PHQ-9 and SSRS

| Way | *Estimate* | *SE* | *CR* | *P* |
| --- | --- | --- | --- | --- |
| SSRS→MUIS | -2.177 | 0.521 | -4.177 | <0.001 |
| SSRS→PHQ-9 | -0.13 | 0.038 | -3.442 | <0.001 |
| MUIS→PHQ-9 | 0.018 | 0.004 | 4.263 | <0.001 |
