# Supplementary material for: Illness uncertainty in individuals with gynecologic and breast cancer: A latent profile analysis and structural equation modeling
Source: PLoS One. 2026 Sep 18;21(9):e0358366. doi: 10.1371/journal.pone.0358366 (PMC13588340; doi:10.1371/journal.pone.0358366)
Supplement: Supplementary file 2 [file pone.0358366.s003.docx]

**General Information**

1. Medical Record Number:

2. Phone Number:

3. Age:

4. Ethnicity:

5. Place of residence: □ Rural □ Urban □ City □ Other

6. Educational Level: □ Primary school or below □ Junior high school □ High school or college diploma □ Bachelor's degree □ Postgraduate degree or above

7. Monthly household income: □ ≤ 2,000 yuan □ 2,001–5,000 yuan □ 5,001–10,000 yuan □ 10,001–20,000 yuan □> 20,000 yuan

8. Marital status: □ Single □ Married □ Divorced □ Widowed

9. Number of children: □ Yes □ No

10. Occupation: □ Employee of government agencies/public institutions/enterprises □ Professional technical personnel (e.g., teachers/dancers/lawyers)

□ Commercial and service sector workers (food service staff, drivers, salespersons, etc.) □ Production personnel in agriculture, forestry, animal husbandry, fisheries, and water resources sectors □ Operators of production and transportation equipment及相关 personnel □ Military personnel □ Students □ Freelancers

□ Retirement □ Other ________

11. Religious belief: □ Yes □ No

12. Primary caregivers during your illness: □ Spouse □ Parents □ Children □ Brothers/sisters □ Caregiver □ Others

13. Disease duration: □ <1 month □ 2–5 months □ 6–12 months □>12 months

14. Time since disease diagnosis when completing the questionnaire: □ Just diagnosed □ 1 month □ 3 months □ 6 months

15. Smoking history: □ Yes □ No

16. History of alcohol consumption: □ Yes □ No

**9-item Patient Health Questionnaire (PHQ-9)**

1. Based on your experience over the past two weeks, please indicate whether you have experienced any of the following conditions and their frequency. Mark the corresponding number next to your selected option (0 for 'never'; 1 for 'several days'; 2 for 'more than half the days'; 3 for 'almost daily').

1. Lack of motivation or interest when performing tasks

□ Not at all □ For several days □ More than half of the days □ Almost every day	□ Not at all □ For several days □ More than half of the days □ Almost every day	□ Not at all □ For several days □ More than half of the days □ Almost every day	□ Not at all □ For several days □ More than half of the days □ Almost every day

2. Experience feelings of depression, frustration, or despair

□ Not at all □ For several days □ More than half of the days □ Almost every day	□ Not at all □ For several days □ More than half of the days □ Almost every day	□ Not at all □ For several days □ More than half of the days □ Almost every day	□ Not at all □ For several days □ More than half of the days □ Almost every day

3. Difficulty falling asleep, restless sleep, or excessive sleep

□ Not at all □ For several days □ More than half of the days □ Almost every day	□ Not at all □ For several days □ More than half of the days □ Almost every day	□ Not at all □ For several days □ More than half of the days □ Almost every day	□ Not at all □ For several days □ More than half of the days □ Almost every day

4. Feeling fatigued or lacking energy

□ Not at all □ For several days □ More than half of the days □ Almost every day	□ Not at all □ For several days □ More than half of the days □ Almost every day	□ Not at all □ For several days □ More than half of the days □ Almost every day	□ Not at all □ For several days □ More than half of the days □ Almost every day

5. Loss of appetite or overeating

□ Not at all □ For several days □ More than half of the days □ Almost every day	□ Not at all □ For several days □ More than half of the days □ Almost every day	□ Not at all □ For several days □ More than half of the days □ Almost every day	□ Not at all □ For several days □ More than half of the days □ Almost every day

6. Feeling extremely inadequate or unsuccessful, or having disappointed oneself or one's family members.

□ Not at all □ For several days □ More than half of the days □ Almost every day	□ Not at all □ For several days □ More than half of the days □ Almost every day	□ Not at all □ For several days □ More than half of the days □ Almost every day	□ Not at all □ For several days □ More than half of the days □ Almost every day

7. Difficulty maintaining focus on tasks, such as when reading a newspaper or watching television.

□ Not at all □ For several days □ More than half of the days □ Almost every day	□ Not at all □ For several days □ More than half of the days □ Almost every day	□ Not at all □ For several days □ More than half of the days □ Almost every day	□ Not at all □ For several days □ More than half of the days □ Almost every day

8. The speed of movement or speech is so slow that others can already perceive it, or conversely, there is excessive restlessness or agitation with frequent pacing that exceeds normal levels.

□ Not at all □ For several days □ More than half of the days □ Almost every day	□ Not at all □ For several days □ More than half of the days □ Almost every day	□ Not at all □ For several days □ More than half of the days □ Almost every day	□ Not at all □ For several days □ More than half of the days □ Almost every day

9、Do you have thoughts of dying or harming yourself in any way? □ Never at all □ For several days □ More than half the time □ Almost every day	Do you have thoughts of dying or harming yourself in any way? □ Never at all □ For several days □ More than half the time □ Almost every day	Do you have thoughts of dying or harming yourself in any way? □ Never at all □ For several days □ More than half the time □ Almost every day	Do you have thoughts of dying or harming yourself in any way? □ Never at all □ For several days □ More than half the time □ Almost every day

**Generalized Anxiety Disorder Scale (GAD-7)**

2. Based on your experience over the past two weeks, please indicate whether you have experienced any of the following conditions and their frequency. Mark the corresponding number next to your selected option (0 = none at all; 1 = several days; 2 = more than one week; 3 = almost daily).

1. Experience tension, anxiety, or urgency

□ Not at all □ For several days □ For more than a week □ Almost every day	□ Not at all □ For several days □ For more than a week □ Almost every day	□ Not at all □ For several days □ For more than a week □ Almost every day	□ Not at all □ For several days □ For more than a week □ Almost every day

2. Failure to cease or control anxiety

□ Not at all □ For several days □ For more than a week □ Almost every day	□ Not at all □ For several days □ For more than a week □ Almost every day	□ Not at all □ For several days □ For more than a week □ Almost every day	□ Not at all □ For several days □ For more than a week □ Almost every day

3. Excessive worry about various matters

□ Not at all □ For several days □ For more than a week □ Almost every day	□ Not at all □ For several days □ For more than a week □ Almost every day	□ Not at all □ For several days □ For more than a week □ Almost every day	□ Not at all □ For several days □ For more than a week □ Almost every day

4. It's very difficult to relax.

□ Not at all □ For several days □ For more than a week □ Almost every day	□ Not at all □ For several days □ For more than a week □ Almost every day	□ Not at all □ For several days □ For more than a week □ Almost every day	□ Not at all □ For several days □ For more than a week □ Almost every day

5. Inability to sit still due to anxiety

□ Not at all □ For several days □ For more than a week □ Almost every day	□ Not at all □ For several days □ For more than a week □ Almost every day	□ Not at all □ For several days □ For more than a week □ Almost every day	□ Not at all □ For several days □ For more than a week □ Almost every day

6. Becoming easily irritated or impatient

□ Not at all □ For several days □ For more than a week □ Almost every day	□ Not at all □ For several days □ For more than a week □ Almost every day	□ Not at all □ For several days □ For more than a week □ Almost every day	□ Not at all □ For several days □ For more than a week □ Almost every day

7. Feeling that something terrible is about to happen and experiencing fear

□ Not at all □ For several days □ For more than a week □ Almost every day	□ Not at all □ For several days □ For more than a week □ Almost every day	□ Not at all □ For several days □ For more than a week □ Almost every day	□ Not at all □ For several days □ For more than a week □ Almost every day

**Medical Uncertainty Scale-MUIS-A (33 items)**

Please read each sentence carefully. First, reflect on the meaning of each statement, then mark "√" in the section that best reflects your recent feelings (on a scale from 'strongly disagree' to 'strongly agree,' scored 1–5; item 15 is not counted toward the total score). The scale ranges from 32 to 160 points, categorized as low-level (32–74.7), moderate-level (74.8–117.4), or high-level (117.5–160); higher scores indicate greater uncertainty about the disease.

I don't know what's wrong with me.

□ Strongly disagree □ Disagree □ Not sure □ Agree □ Strongly agree	□ Strongly disagree □ Disagree □ Not sure □ Agree □ Strongly agree	□ Strongly disagree □ Disagree □ Not sure □ Agree □ Strongly agree

I have many questions that don't have answers.

□ Strongly disagree □ Disagree □ Not sure □ Agree □ Strongly agree	□ Strongly disagree □ Disagree □ Not sure □ Agree □ Strongly agree	□ Strongly disagree □ Disagree □ Not sure □ Agree □ Strongly agree

I am uncertain how my condition will evolve.

□ Strongly disagree □ Disagree □ Not sure □ Agree □ Strongly agree	□ Strongly disagree □ Disagree □ Not sure □ Agree □ Strongly agree	□ Strongly disagree □ Disagree □ Not sure □ Agree □ Strongly agree

I am not sure to what extent I will experience pain (or discomfort).

□ Strongly disagree □ Disagree □ Not sure □ Agree □ Strongly agree	□ Strongly disagree □ Disagree □ Not sure □ Agree □ Strongly agree	□ Strongly disagree □ Disagree □ Not sure □ Agree □ Strongly agree

The medical staff did not provide a sufficiently clear explanation of my condition.

□ Strongly disagree □ Disagree □ Not sure □ Agree □ Strongly agree	□ Strongly disagree □ Disagree □ Not sure □ Agree □ Strongly agree	□ Strongly disagree □ Disagree □ Not sure □ Agree □ Strongly agree

I am fully aware of the purpose of each treatment.

□ Strongly disagree □ Disagree □ Not sure □ Agree □ Strongly agree	□ Strongly disagree □ Disagree □ Not sure □ Agree □ Strongly agree	□ Strongly disagree □ Disagree □ Not sure □ Agree □ Strongly agree

When I experience pain (or discomfort), I understand the significance of such sensations for my condition.

□ Strongly disagree □ Disagree □ Not sure □ Agree □ Strongly agree	□ Strongly disagree □ Disagree □ Not sure □ Agree □ Strongly agree	□ Strongly disagree □ Disagree □ Not sure □ Agree □ Strongly agree

I cannot predict what changes may occur in my condition.

□ Strongly disagree □ Disagree □ Not sure	□ Agree □ Strongly agree	□ Agree □ Strongly agree

I cannot predict changes in physical symptoms.

□ Strongly disagree □ Disagree □ Not sure	□ Agree □ Strongly agree	□ Agree □ Strongly agree

I understand every detail that the medical staff explained to me.

□ Strongly disagree □ Disagree □ Not sure	□ Agree □ Strongly agree	□ Agree □ Strongly agree

The words spoken by the doctor may carry multiple distinct meanings.

□ Strongly disagree □ Disagree □ Not sure	□ Agree □ Strongly agree	□ Agree □ Strongly agree

I can predict how long I will be ill.

□ Strongly disagree □ Disagree □ Not sure	□ Agree □ Strongly agree	□ Agree □ Strongly agree

The treatment I received was highly complex and difficult to comprehend.

□ Strongly disagree □ Disagree □ Not sure	□ Agree □ Strongly agree	□ Agree □ Strongly agree

I am uncertain whether the treatment I have received or the medications I have taken are effective.

□ Strongly disagree □ Disagree □ Not sure	□ Agree □ Strongly agree	□ Agree □ Strongly agree

The hospital employs a variety of staff members, and I am not aware of their specific responsibilities.

□ Strongly disagree □ Disagree □ Not sure	□ Agree □ Strongly agree	□ Agree □ Strongly agree

Since the disease progression cannot be predicted, I am unable to plan for the future.

□ Strongly disagree □ Disagree □ Not sure	□ Agree □ Strongly agree	□ Agree □ Strongly agree

My condition is fluctuating, with periods of improvement and exacerbation.

□ Strongly disagree □ Disagree □ Not sure	□ Agree □ Strongly agree	□ Agree □ Strongly agree

I am unsure how to manage my daily life after discharge.

□ Strongly disagree □ Disagree □ Not sure	□ Agree □ Strongly agree	□ Agree □ Strongly agree

I consulted numerous individuals, and received varying accounts regarding my condition.

□ Strongly disagree □ Disagree □ Not sure	□ Agree □ Strongly agree	□ Agree □ Strongly agree

I don't know what else might happen to me in the future.

□ Strongly disagree □ Disagree □ Not sure	□ Agree □ Strongly agree	□ Agree □ Strongly agree

I am generally aware of whether the patient's physical condition on that day is good or poor.

□ Strongly disagree □ Disagree □ Not sure	□ Agree □ Strongly agree	□ Agree □ Strongly agree

My test results are inconsistent.

□ Strongly disagree □ Disagree □ Not sure	□ Agree □ Strongly agree	□ Agree □ Strongly agree

The efficacy of the treatment I have received has not yet been confirmed.

□ Strongly disagree □ Disagree □ Not sure	□ Agree □ Strongly agree	□ Agree □ Strongly agree

It's quite unclear how long it will take before I can take care of myself independently.

□ Strongly disagree □ Disagree □ Not sure	□ Agree □ Strongly agree	□ Agree □ Strongly agree

I can roughly predict the progression of my condition.

□ Strongly disagree □ Disagree □ Not sure	□ Agree □ Strongly agree	□ Agree □ Strongly agree

Due to therapeutic considerations, the actions that are permissible and those that are prohibited continuously vary.

□ Strongly disagree □ Disagree □ Not sure	□ Agree □ Strongly agree	□ Agree □ Strongly agree

I am certain that neither the physician nor the nurse will identify any additional abnormalities in me.

□ Strongly disagree □ Disagree □ Not sure	□ Agree □ Strongly agree	□ Agree □ Strongly agree

The treatment I am receiving has a high success rate.

□ Strongly disagree □ Disagree □ Not sure	□ Agree □ Strongly agree	□ Agree □ Strongly agree

The physician did not provide me with a definitive diagnosis.

□ Strongly disagree □ Disagree □ Not sure	□ Agree □ Strongly agree	□ Agree □ Strongly agree

I can predict physical discomfort and know what will improve or worsen.

□ Strongly disagree □ Disagree □ Not sure	□ Agree □ Strongly agree	□ Agree □ Strongly agree

When I need assistance, the nursing staff can provide timely support.

□ Strongly disagree □ Disagree □ Not sure	□ Agree □ Strongly agree	□ Agree □ Strongly agree

The severity of my condition is now well established.

□ Strongly disagree □ Disagree □ Not sure	□ Agree □ Strongly agree	□ Agree □ Strongly agree

I can understand the medical staff's explanations regarding the treatment.

□ Strongly disagree □ Disagree □ Not sure	□ Agree □ Strongly agree	□ Agree □ Strongly agree

**Social Support Rating Scale (SSRS) (10 items)**

5. Please read the following sentences and mark "√" in the column that best reflects your actual situation.

1. How many close friends do you have who can provide support and help? (Select one only) ()

There isn't one.

② 1–2.

③ 3–5.

④ Six or more.

2. Over the past year, you have: (Select one only) ()

① Stay away from family members and live alone in a separate room.

② Frequent changes in residence, spending most of the time living with strangers.

③ Living with classmates, colleagues, or friends.

④ Live with family.

3. You and your neighbor: (Select one only) ()

① They never show any concern for each other; they merely have a superficial acquaintance.

② May show some concern when encountering difficulties.

③ Some neighbors care a lot about you.

④ Most of your neighbors care about you.

4. You and your colleagues: (Select one only) ()

① They never show any concern for each other; they merely have a superficial acquaintance.

② May show some concern when encountering difficulties.

③ Some colleagues are very concerned about you.

④ Most of your colleagues care deeply about you.

5. Support and care received from family members: (Check "√" in the appropriate box) (Select only one option) ()

|  | ① not have | ② precious few | ③ same as | ④ Full support |
| --- | --- | --- | --- | --- |
| 5. Spouses (Lovers) |  |  |  |  |
| 6. Parents |  |  |  |  |
| 7. Children |  |  |  |  |
| 8. Brothers and sisters |  |  |  |  |
| 9. Other members (e.g., sisters-in-law) |  |  |  |  |

10. In the past, the sources of financial assistance or practical help you received when facing urgent or difficult situations were: ()

①No source identified.

②มาจาก the following sources (multiple selections allowed): ()

A. Spouse. B. Other family members. C. Friends. D. Relatives. E. Colleagues. F. Workplace. G. Official or semi-official organizations such as party groups, youth leagues, and trade unions. H. Non-official organizations such as religious or social groups. I. Others.

11. In the past, when you faced difficulties or urgent challenges, the sources of comfort and support you received included: ()

①No source identified.

②มาจาก the following sources (multiple selections allowed): ()

A. Spouse. B. Other family members. C. Friends. D. Relatives. E. Colleagues. F. Workplace. G. Official or semi-official organizations such as party groups, youth leagues, and trade unions. H. Non-official organizations such as religious or social groups. I. Others.

12. How you usually talk about your troubles: (Select one only) ()

① Never tell anyone about it.

② Share only with 1–2 people who have an extremely close relationship.

③ Tell them when a friend asks directly.

④ Proactively express your concerns to gain support and understanding.

13. How you seek help when facing difficulties: (Select one only) ()

① Rely solely on oneself and refuse assistance from others.

② Rarely asks others for help.

③ Sometimes ask others for help.

④ When facing difficulties, one should frequently seek assistance from family members, relatives, or organizations.

14. For group organizations (e.g., party organizations, religious groups, trade unions, student unions, etc.) conducting activities, please select one option: ()

①Never participate.

② Participate occasionally.

③ Participate regularly.

④ Actively participate and engage in initiatives.
